# Cheoplasty

**Published:** 1857-12

**Authors:** 


### CHEOPLASTY.

Among the wonderful improvements of the present age, few things have received greater advances than the art of puffing. This is the day of humbug, and their recommendations seem to be obtained with increased facility from those occupying high civil and military stations. Most of our newspapers abound in puffs of quack medicines, not unfrequently given by physicians, who should know that diagnosis should precede prescriptions, and that the latter should be known, not taken at random.

The most available puffs, are, no doubt, those from clergymen (headed sometimes by "Hear what the preacher saith") who ought to understand that it is not their legitimate business to dabble in nostrums, degrading their calling by aiding in imposition on the credulity of the public. The Apostles were sent forth endowed with power to heal the sick, and ordered to use it as proof above cavil, that they were Jehovah's commissioned agents to proclaim His gospel. Subsequently the command to them (under which their successors act) was to preach and teach all nations the way of salvation. The preacher's vocation is to heal, not the physical, but the spiritual diseases of men.

The invention denominated cheoplasty has been singularly fortunate in its puffing arrangements. When the clarion notes of its praise first broke upon our ears, we were taken all aback. It was possible that towards the eastern boundary of our country, where the tide ebbs and flows twice every twenty-four hours, and the god of day greets its denizens half an hour sooner than in the central regions—new light had burst forth. We were almost ready to believe that, as far as artificial dentures were concerned, the glory of gold was departing, and that it must cave in to pewter; and then the draft on time, patience and purse were so lessened, that it just suited these fast times—but presently came thoughts of gutta percha and other patents, and the idea that perhaps some of those who recommended cheoplasty might be mistaken for want of sufficient experiment and observation; and that others, although very good men, certified in accordance with a game well understood in certain eastern localities, yclept, "You tickle me, and I'll tickle you."

Ere long the adulations from the East were echoed, rather faintly to be sure, from the center of our country. A very clever member of our profession in St. Louis made a pilgrimage to Baltimore, where, treated like a veritable devotee to cheoplasty, he was favorably impressed with it; and, with very limited opportunity to watch practical results, returning to his home in time to be present at a meeting of the Western Dental Society, and was one of a committee which prepared a favorable report, which was adopted. But the result of sober second thought, aided by ample experience, is, that cheoplasty is not used by any one in St. Louis (so I understand) or Cincinnati.

In the American Journal of Dental Science for the present month, appear two articles, of stamp similar to nostrum puffing generally, highly laudatory of cheoplasty. In reference to the only invention of consequence to it, (most of the other steps in the process having been previously invented by others), the writer says: "The patentee has wisely withheld from his patrons, the formula by which his metal is compounded; and, I think the reason which he assigns is satisfactory—that the metal would certainly degenerate if indiscriminately compounded. But I am willing to believe that it is at least as pure as block tin; and if so, time has already proved to me that it may be worn in the mouth with impunity."

Webster's first definition of "pure" is "separate from all heterogenous or extraneous matter; clean, free from mixtuı e, as pure water, pure clay, pure sand, pure air, pure silver and gold." But Dr. Shepherd is willing to believe that tin combined with small proportions of bismuth, cadenium, silver, and antimony, is "as *pure* as block tin!" There is no *purity* about it; it is only a base compound, and if there is any truth inculcated in the standard works on metallurgy, the tendency of the different metals to oxidize, is increased by their combination.

It would seem from the trivial cost of the metals for the compound—a transcript of the formula can be readily obtained from the patent office—that the inducement to degenerate it must be very weak.

The second article, on "Cheoplasty and its Results," opens magniloquently about the world's heroes, with garlands of fame, their pathway strewn with flowers, and the benefactor of his fellow man standing pre-eminently above them all. Then follows, "To the author of cheoplasty the highest meed of praise is due; he has nobly entered into the broad arena of science, to battle in its holy cause, and in despite of the sneers of the unbelieving, or the malicious efforts of enemies, has, in his new process, won for himself imperishable honor, and brought dental mechanism to a high state of perfection." Next in course is the offering up the incense of a grateful heart to the inventor, and the hope expressed that the time will soon come when the "whole profession will join in swelling the notes of praise to the author of this invaluable compound." This, without patent-rights, would be glory enough for most men.

Again, "Through the gloomy mist that conceals the future from the present, we can plainly discern that approaching period, when this method shall supercede all others." Keen perceptives has Dr. Brown to "*plainly discern*" what gloomy mist "*conceals*" from common eyes.

The writer gives among its advantages, accuracy of adaptation, ease and safety of repairs, and "then again possessing the same non-corrosive property as gold!!!" No more objections, of course, to cement in the great anti amalgam journal, when it contains such statements as this without protest. How appropriate is the remark of the writer in this article: "This is truly the age of progress;" "New light is now shining in dark places." Tin, cadenium, etc., combined, as non-corrosive as gold! Finish a piece of Dr. Blandy's cheoplasty nicely, and place it on a shelf in any laboratory during ten days, and it will exhibit evidence of corrosion similar to that of an old pewter spoon in a neglected scullery.

The same highfalutin, *truthful* strain is continued: "In this work there are no spaces left in which food and secretions can lodge, hence for comfort, cleanliness and utility, it stands unrivalled and unsurpassed." Any thing as to "no spaces left" superior to Prof. Allen's continuous gum, the gutta percha, or tin work of years past? This is new light in dark places with a vengeance.

To substantiate all this the writer gives us several letters, evincing by his laudatory remarks of their authors an intimate acquaintance with quack almanac literature, and the noble science of puffing. We learn from one of the certificates that, in his mouth, gold and platina "have a very disagreeable black incrustation," while the cheoplastic compound remains "pure and lustrous."

Finally, by way of a clencher, we have a letter from a "distinguished minister." With such indubitable proof that cheoplasty is perfectly free from all tendency to discoloration, and of "its perfect tastelessness and purity," let all unbelievers and objectors fall into the ranks, throw up their caps and "join in swelling the notes of praise to the author of this invaluable compound," or, chop-fallen, hide their diminished heads at once and forever.

NOVICE.

OCTOBER 29th, 1857.

